# Postgraduate education in nutrition in south Asia: a huge mismatch between investments and needs

**DOI:** 10.1186/1472-6920-14-3

**Published:** 2014-01-07

**Authors:** Shweta Khandelwal, Tanusree Paul, Lawrence Haddad, Surbhi Bhalla, Stuart Gillespie, Ramanan Laxminarayan

**Affiliations:** 1 Public Health Foundation of India, 14 Community Centre, Panchsheel Park, New Delhi 110016, India; 2 Institute of Development Studies, Sussex, Brighton BN1 9RH, UK; 3 International Food Policy Research Institute, 2033 K St, NW, Washington DC 20006-1002, USA

**Keywords:** Public health nutrition, Higher Education, Capacity building, South Asia, Curriculum

## Abstract

**Background:**

Despite decades of nutrition advocacy and programming, the nutrition situation in South Asian countries is alarming. We assume that modern training in nutrition at the post graduate level is an important contributor to building the capacity of individuals to think and act effectively when combating undernutrition. In this context, this paper presents a regional situation analysis of master’s level academic initiatives in nutrition with a special focus on the type of programme we think is most likely to be helpful in addressing undernutrition at the population level: Public Health Nutrition (PHN).

**Methods:**

This situational analysis of Masters in nutrition across South Asian countries viz. India, Pakistan, Bangladesh, Sri Lanka, Afghanistan, Maldives, Nepal, Bhutan was conducted using an intensive and systematic Internet search. Further, detailed information was extracted from the individual institute websites and library visits.

**Results:**

Of the131 master’s degree programmes we identified one that was in PHN while another 15 had modules in PHN. Most of these universities and institutions were found in India with a few in Bangladesh and Sri Lanka. In the rest of the countries, neither nutrition nor PHN emerged as an academic discipline at the master’s level. In terms of eligibility Indian and Sri Lankan programmes were most inclusive, with the remaining countries restricting eligibility to those with health qualifications. On modules, no country had any on nutrition policy or on nutrition’s interactions with agriculture, social protection, water and sanitation or women’s empowerment.

**Conclusion:**

If a strong focus on public health nutrition is key to reducing undernutrition, then the poor availability of such courses in the region is cause for concern. Nutrition master’s courses in general focus too little on the kinds of strategies highlighted in the recent Lancet series on nutrition. Governments seeking to accelerate declines in undernutrition should incentivize the delivery of postgraduate programmes in nutrition and Public Health Nutrition (PHN) that reflect the modern consensus on priority actions. In the absence of PHN type programmes, the competence to scale up nutrition capacity is likely to be impaired and the human potential of millions of infants will continue to be squandered.

## Background

Approximately 165 million children under 5 are stunted and more than half of them live in South Asia [1]. India alone has 62 million stunted children. Undernutrition more generally is the underlying cause of 45 percent of all child deaths, and the prevention of stunting would add at least 11% to GDP in South Asia [2]. Improving the growth and development of children accelerates the economic growth and development of nations [3-5].

Much has changed about the way we think about undernutrition reduction during the last decade. We now understand that effective action must address determinants at the immediate, underlying and basic levels. The Lancet Maternal and Child Nutrition Series (2013) calls for a balanced approach to investing in and scaling up interventions that are nutrition specific, nutrition sensitive and create an enabling environment for undernutrition reduction [3]. Financial resources are a key part of this investment, but so too are investments in human resources. Many such investments will be made with public funds, but many will be made through private investment. The funds that are paid to cover fees for university courses in nutrition represent a major investment, some public some private. How aligned are the priorities of these courses with the public health nutrition (PHN) priorities of the countries of South Asia?

This paper represents a first step towards the answer to this question. The paper describes the number and types of post graduate nutrition programmes found in 8 South Asian countries [5].

The importance of the quality of nutrition professionals for effective nutrition programme delivery has been repeatedly highlighted in the literature [6-8]. Having said that, there are two important questions that need to be clarified at this juncture- why are we focusing on PHN and graduate training? Success stories of effectively addressing malnutrition among children across the world reveals that community based interventions integrated with existing health services, that developing the capacities of staffs, stakeholders and frontline workers, to integrate nutrition within health and with other sectors and services and to increase nutrition surveillance and monitoring at the community level are some of the most important steps adopted by countries like Ethiopia, Nepal, Democratic Republic of Congo, Maharashtra- India, Sri Lanka, United Republic of Tanzania to name a few [9]. Compared to nutrition and dietetics programmes, PHN courses tend to be more population and community focused as well as more programme and policy focused. The focus on reason behind focusing on postgraduate training assumes that most of these individuals go on to become nutrition professionals. As such they are only one component of nutrition capacities at all levels- viz. system, organizational, and individual and at many levels of qualification, but we feel they help set the norms and rules that others work to and hence they have an important transformational function [10]. So we highlight the need for accreditation and formal training in form of postgraduate education in PHN as a small yet potentially transformational step towards strengthening the PHN capacity in South Asia. We propose that postgraduate training in PHN will help professionals understand the importance of locating nutrition in a broader development context, helping them see the need for a balance between nutrition specific, nutrition sensitive and enabling environment actions to reduce undernutrition in a sustained manner.

The undernutrition problems facing the 8 countries in the region are profound. Table 1 summarises the stunting and wasting rates of children under 5 in these countries. India, Pakistan and Bangladesh are home to 98% of the stunted children in the region, while India alone is home to 78%.

**Table 1:** Undernutrition among children under 5 in South Asia

**Country**	**Stunting rate**	**Number of stunted children under 5 (millions)**	**Wasting rate**
Sri Lanka	17	0.33	15
Maldives	19	0.005	11
Bhutan	34	0.023	6
Bangladesh	41	6	16
Nepal	41	1.4	11
Pakistan	44	10	15
India	48	62	20
Afghanistan	59	No data	9

Source: UNICEF 2013. Improving child nutrition. The achievable imperative for global progress.

## Methods

We conducted a search for all nutrition related post graduateprogrammes in the 8 South Asian countries: Afghanistan, Pakistan, India, Sri Lanka, Nepal, Bhutan, Bangladesh and Maldives. We used a combination of search words and phrases within freely available search engines such as Google, Google Scholar and PubMed: ‘public health nutrition colleges’, ‘masters in public health nutrition’, ‘nutrition’, ‘nutrition colleges’, ‘nutrition institutes’, ‘home science’, ‘community nutrition’, ‘therapeutic nutrition’, ‘food and nutrition’, ‘diploma or certificate course in nutrition’, ‘diploma and certificate course in public health’ in the respective countries. In this paper the term ‘courses’ refers to the academic programmes while ‘module’ refers to the specific segments within the courses.

After acquiring preliminary information on the names of institutes/colleges offering academic degrees in nutrition we collected detailed information such as: contact address, phone numbers, courses, fee structure, batch/class size, and eligibility criteria from institute/university’s websites. Electronic libraries and national repositories of information on Nutrition and PHN education were also looked into. The details were compiled and entered into a matrix which has been provided as an Excel file (see Additional file 1). Additional information pertaining to health and nutrition related outcomes of the 8 countries was derived from various country pages of the World Health Organization and World Bank.

## Results and discussion

The situation analysis attempted to map the postgraduate academic initiatives in nutrition with a special focus on Public Health Nutrition in South Asia. Emphasis in the descriptive analysis is given to academic levels, specialization, courses/modules, mode of delivery, duration and eligibility criteria for the courses. Details for each country have been described in a systematic fashion. For countries with little available detailed information, we have aggregated the data.

### India

A master’s in Nutrition was offered in about 112 institutes, mostly being subsumed under departments of Home Science and Health Sciences. The various specializations were Food and Nutrition, Home Science, Nutrition and Dietetics, Food Technology and Management, Nutritional Sciences, Clinical Nutrition, Applied nutrition, Dietetics, Family and Community Medicine, Community Health and Nutrition, Food Science and Technology, Clinical Nutrition and Dietetics, Food Service Management, Food Science, Food Analysis and Quality Assurance, Food Biotechnology. These were mostly full time courses. PHN (at the master’s level) was offered in 13 institutes and universities across India, although opportunities were confined to modules or post graduate diploma programmes rather than as a full-fledged course. Only in one –Baroda - was it offered as a master’s course.

The master’s courses were offered for a period of 2 years while the postgraduate diploma is for 1 year. Course fees ranged from less than 1000 USD to 3000 USD. Eligibility requirements include holding a relevant Bachelors degree such as a B.Sc. (Bachelor in Science) in Agriculture/Horticulture; B.Tech. (Bachelor in Technology) in Agricultural Engineering/Dairying of any State Agricultural University/National Dairy Research Institute; BVSc& AH (Bachelor of Veterinary Science and Animal Husbandry), BSSc. (Bachelor of Social Science), and a B.Sc. (Bachelor of Science or Food Science and Technology).

### Pakistan

In Pakistan, nutrition as an academic master’s discipline was found to be housed under departments of public health, departments of agriculture, and departments of human nutrition. The courses found were an M.Sc. in Nutrition (n = 1), an M.Sc. in Community Health and Nutrition (n = 1), M.Sc. in Food Science and Nutrition (n = 2), an M.Sc. in Food Technology (n = 1) and an M.Sc. in Home Economics (Food and Nutrition) (n = 1). The courses were regular and full time with English as the medium of instruction. Public Health Nutrition did not emerge in a single institute/university, not even as specialized modules within a programme.

The master’s courses were offered for duration of 1–2 years with approximate course fee ranging between 2000–3000 USD. Candidates with 4 years bachelor’s degree in a discipline related to health sciences or statistics from an accredited institution, approved by Higher Education Commission of Pakistan; MBBS (Bachelor of Medicine & Bachelor of Surgery), BDS (Bachelor of Dental Surgery); B. Pharmacy (Bachelor’s in Pharmacy) or M. Pharmacy (Masters in Pharmacy); B.Sc. Nursing (Bachelor of Sciences in Nursing); Medical technologists having four years education after F.Sc (Faculty of Science); DVM (Doctor of Veterinary Medicine); those with a master’s Degree in a relevant subject such as Anthropology, Business Administration, Human Nutrition, Microbiology, Physiology, Psychology, Sociology and Zoology were eligible to apply for the nutrition master’s courses.

### Bangladesh

In Bangladesh, we found four institutes or universities that offered a Masters in Public Health. Two offered PHN as a course and one offered Nutrition as a major subject. There was also a certificate course on Applied Dietetics and Food Technology where nutrition was a focus.

The master’s programmes were of 1–2 years duration. Candidates with a Bachelors degree in health related subjects or allied sciences; bachelor and/or master’s degree(s) in medicine, nursing, midwifery, health sciences, social sciences or other sciences were eligible. Course fees were not available for most of the universities except the BRAC University, where the fee was 20,000 USD that covered tuition fee (international and national faculty), logistics, food, local transportation, support for field work activities, course materials and supplies for the entire duration of the course.

### Nepal

In Nepal, there was little explicit focus on Nutrition as a separate academic discipline with the latter being mostly housed under departments of Public Health. Thus our focus was on Masters in Public Health with Nutrition as a module. Altogether four institutes or universities offered Masters in Public Health with Nutrition as a module (n = 4). In only one university did Nutrition emerge as a distinct discipline within a master’s, being offered as Food and Nutrition with a focus on community nutrition, dietetics, and applied nutrition. In the remaining Masters programmes nutrition was a focus of one section of a family health module (n = 1), and was subsumed under “child health” in Environmental Health and Disease Control (n = 1) and under ‘nutrition and health’ in Family Health (n = 1).

In all the institutes/universities, the courses were run mostly as regular and full time with a duration of 1–2 years. The medium of instruction was English. Candidates with a Bachelor’s Degree in Health Sciences (MBBS, Public Health and Bachelor of Nursing/B.Sc. Nursing), Pharmacy, Radiography, Health Lab and veterinary Medical Sciences from accredited institutions with 1–3 years work experience in concerned health related fields were eligible to apply for the course.

### Sri Lanka

In Sri Lanka, nutrition related master’s degrees were offered in three Universities in form of M.Sc. in Human Nutrition (n = 1), M.Sc. in Food and Nutrition (n = 1), and a M.Sc. in Applied Epidemiology (n = 1). Those were housed either under the academic departments of Nutrition or Food Science and Technology. PHN was offered as a module under one course, the M.Sc. in Human Nutrition. These were regular full time courses with English as the medium of instruction.

The master’s courses offered were for 1.5 to 2 years. The approximate course fee ranged between 1000–1700 USD. Candidates with BVSc (Bachelor of Veterinary Science)/MBBS/B.Sc. or equivalent certification; Bachelors degree in Agriculture, Science or related disciplines from a recognized institute of higher education, Bachelors degree in Science of Medicine were eligible for the courses.

### Bhutan, Maldives and Afghanistan

We found little information on Bhutan, Afghanistan and Maldives. In Afghanistan, there was no mention of nutrition or PHN within academic courses. Courses on Public Health were offered within Medical Departments in most universities.

In Bhutan, there was only one institute that offered a module on nutrition and dietetics in the Basic Science Department as part of a Bachelors degree programme. There were no master’s programmes in either Nutrition or PHN. In Maldives, no institute/university offered any course on nutrition, at the undergraduate or postgraduate level.

Table 2 summarises the findings for Masters Programmes in Nutrition. India had by far the largest number of master’s programme. The total population per courses was the highest in Bangladesh followed by Pakistan and Nepal. On the other hand, the ratio between the total number of undernourished children to the total number of courses was the highest in Pakistan followed by Bangladesh and Nepal. On eligibility, in India, and to some extent in Sri Lanka, the courses are not restricted to graduates in medicine-- those from other disciplines like Agriculture and Horticulture are also invited to apply. In all the other countries, the eligibility criteria are strictly restricted to medical graduates and those in Health Sciences.

**Table 2:** Details of master’s courses in nutrition in South Asian countries

**Countries**	**Number of courses**	**Eligibility criteria**	**Total population per course**	**Number of undernourished children per course**	**Fee structure (approximately)**	**Duration**
India	112	B.Sc. in Agriculture/Horticulture of 4 year duration; B.Tech. in Agricultural Engineering/Dairying of any State Agricultural University/National Dairy Research Institute; BVSc & AH, BSSc, BSC (Food Science and Technology).	10,805,298	553,571	1000-3000 USD	2 years
Pakistan	6	4 years bachelor’s degree in a discipline related to health sciences or statistics from an accredited institution, approved by Higher Education Commission of Pakistan; MBBS (Bachelor of Medicine & Bachelor of Surgery), BDS (Bachelor of Dental Surgery); B. Pharmacy (Bachelor’s in Pharmacy) or M. Pharmacy (Masters in Pharmacy); B.Sc. Nursing (Bachelor of Sciences in Nursing); Medical technologists having four years education after F.Sc; DVM (Doctor of Veterinary Medicine); Master’s Degree in a relevant subject such as Anthropology, Business Administration, Human Nutrition, Microbiology, Physiology, Psychology, Sociology and Zoology	29,833,333	1,666,667	2000-3000 USD	1-2 years
Bangladesh	4	B.SC in health related subjects for M.SC in Nutrition Bachelor and/or Masters degree(s) in medicine, nursing, midwifery, health sciences, social sciences or other sciences; MBBS or Bachelors in allied sciences	37,173,033	1,500,000	20,000 USD (in 1 university, details not available for others)	1-2 years
Nepal	1	Bachelor’s Degree in Health Sciences (MBBS, Public Health and BN/B.Sc. Nursing), Pharmacy, Radiography, Health Lab and veterinary Medical Sciences from accredited institutions with 1–3 years work experience in concerned health related field	27,474,000	1,400,000	Not available	1-2 years
Sri Lanka	3	BVSc/MBBS/BSC or equivalent certification, Bachelor s degree in Agriculture, Science or related disciplines from a recognized institute of higher education, Bachelors degree in Science of Medicine	7,032,667	110,000	1000-1700 USD	1.5-2 years.

Source: compiled by authors.

The content of curricula is an important factor which determines the efficacy and capacity of the intended workforce. In this context, the use of competency based approaches have been widely advocated since such approaches provide an architecture for workforce development by codifying the knowledge, skills and attitudes necessary to effectively practice [11]. Although a detailed competency based curricula analysis is beyond the scope of this paper, we hereby present an exploratory overview of the courses offered in the South Asian countries under the master’s programme.

**Figure 1:**
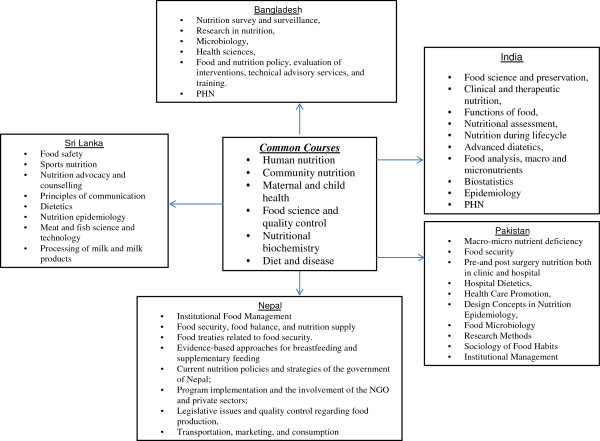
Courses offered under master’s programme in nutrition.

Different countries had varying thematic focus within the nutrition curriculum, as shown in Figure 1. In Bangladesh, it was nutrition survey and surveillance, research in nutrition, food sciences, microbiology, health sciences, food and nutrition policy, PHN, evaluation of interventions, technical advisory services, and training. In Sri Lanka and Nepal- human nutrition, community nutrition, public health nutrition and gender studies, food science and quality control, nutritional biochemistry, diet and disease are the common courses. Additionally, institutional food management was a unique course taught in Nepal while food safety, sports nutrition, nutrition advocacy and counseling, principles of communication, dietetics, nutrition, PHN and epidemiology were some of the courses that were taught in Sri Lanka. In India, the nutrition related courses included nutritional biochemistry, food science and preservation, maternal and child nutrition, clinical and therapeutic nutrition, functions of food, nutritional assessment, community nutrition, nutrition during lifecycle, advanced dietetics, food analysis, PHN, macro and micronutrients. In terms of the common courses we note the absence of nutrition policy and the interactions of nutrition with other sectors such as agriculture, social protection, women’s empowerment and water and sanitation. These are all vital to the scaling up of nutrition actions.

Out of a total of 131 master’s programmes, only 16 could be found that contained modules in PHN and these are listed in Table 3. There was only one Masters Programme called Public Health Nutrition (at the University of Baroda) This lack of presence of PHN is worrying given that PHN teaching is most aligned, at least in theory, with the new Lancet 2013 recommendations on how to reduce undernutrition.

**Table 3:** Universities/Institutions Offering PHN as a Course/Module at a postgraduate level in South Asia

**S. no.**	**Name of colleges**	**Country**	**Name of course**
1	MS university of Baroda	India	Masters of Public Health Nutrition
2	University of Calicut	India	M.Sc. in Family & Community Medicine
3	Vidyasagar University	India	M.Sc. in Dietetics & Community Nutrition Management
4	National Institute of Nutrition	India	M.Sc. in Applied Nutrition
6	University of Delhi	India	M.Sc. in Home Science and Food and Nutrition
7	Government Bilasa Girls’ PG College, Bilaspur	India	M.Sc. in Home Science
8	SNDT College, Pune	India	M.SC in Food Science and Nutrition
9	Indira Gandhi national open University, Delhi	India	M.SC in Dietetics & Food Service Management
10	University of Pune	India	M.Sc. in Nutritional Sciences (focus on PHN)
11	St. Teressa College, Kochi	India	M.Sc. in Home Science
12	Nirmala Niketan College of Home Science, Mumbai	India	M.Sc. in Home Science
13	Osmania University College for Women, Koti	India	M.Sc. in Nutrition and Dietitics
14	Post Graduate Institute of Medicine, Colombo	Srilanka	M.Sc. in Human Nutrition
15	BRAC University, Dhaka	Bangladesh	Master in Public Health
16	North South University, Dhaka	Bangladesh	Master in Public Health

Source: compiled by authors.

## Conclusions

The analysis reveals two main things:

First, India has more master’s programmes than other countries in the region. After taking total population sizes and undernutrition figures into account, Pakistan and Bangladesh appear particularly poorly served by post graduate nutrition offerings.

Second, PHN appears only once out of 131 nutrition master’s from the region. PHN is to be found in 15 of the remaining 130 master’s programmes, but only as modules in other courses. India seems to be at the forefront of PHN in the region, but with only one Masters in PHN, the room for improvement is vast.

This lack of nutrition programmes outside of India and the lack of PHN progarmmes in all countries is a real problem. In part, because of the high and persistent undernutiriton rates in the 8 countries, their governments are becoming more committed to action to address undernutrition. Many have, for example, joined the Scaling Up Nutrition Movement (SUN) -- with India a notable exception–and have developed nutrition plans and committed to key nutrition targets [2,12-15]. However, such activity needs to be backed up by the production of trained and sensitized individuals within the policy and practice communities who can play leadership roles in transforming the nutritional ideas and practices. Nutrition professionals who are trained exclusively in home science, or come solely from medical, nursing or dietetic backgrounds are unlikely to be appropriately trained to understand nutrition challenges holistically, especially the social and community dimensions of nutrition problems. Hence, there is an increasing need for training in PHN competencies [16] to expand the horizons of the nutrition professionals from a clinical to a societal approach. This would help them to more effectively address the nutrition problems and challenges at the community, programmatic and public policy levels.

In conclusion, we feel that a strong focus on public health nutrition is key to reducing undernutrition. Governments seeking to accelerate declines in undernutrition should incentivize the delivery of postgraduate programmes in nutrition, and in particular, Public Health Nutrition. In the absence of PHN type programmes, the competence to scale up nutrition capacity is likely to be impaired and the human potential of millions of infants will be squandered.

## Competing interests

The authors declare that they have no competing interests.

## Authors’ contributions

SK conceptualized the study, framed the research design and drafted the manuscript. TP undertook acquisition of data, analysis and interpretation of data, also contributed in drafting the manuscript. LH provided critical inputs in manuscript finalisation. SB undertook acquisition of data. SG provided critical inputs in manuscript finalisation. RL provided critical inputs at all stages of manuscript preparation. All authors have read and approved the manuscript.

## Pre-publication history

The pre-publication history for this paper can be accessed here:

http://www.biomedcentral.com/1472-6920/14/3/prepub

## Supplementary Material

Additional file 1 **Details of masters in nutrition courses in South Asia.** The Additional file 1 lists out the names of colleges and universities with their respective geographical locations and contact details. It also presents the details of master’s courses pertaining to name of degree awarded, specialisations, affiliations, eligibility criteria, mode of delivery and course contents. Click here for file
